# Gender-specific HIV and substance abuse prevention strategies for South African men: study protocol for a randomized controlled trial

**DOI:** 10.1186/s13063-018-2804-3

**Published:** 2018-08-03

**Authors:** Mary Jane Rotheram-Borus, Mark Tomlinson, Andile Mayekiso, Jason Bantjes, Danielle M. Harris, Jacqueline Stewart, Robert E. Weiss

**Affiliations:** 1 Department of Psychiatry & Biobehavioral Sciences, Semel Institute, University of California Los Angeles, 10920 Wilshire Blvd., Suite 350, Los Angeles, California 90024 USA; 2 Department of Psychology, Stellenbosch University, Private Bag X1 Matieland, Stellenbosch, 7602 South Africa; 3 Department of Biostatistics, Fielding School of Public Health, University of California Los Angeles, Los Angeles, CA 90095-1772 USA

**Keywords:** Men’s HIV risk, Soccer, Drug abuse, Alcohol abuse, Gender-specific interventions, HIV prevention for men, HIV testing, HIV prevention strategies, Social determinants of HIV

## Abstract

**Background:**

Young men in South Africa face concurrent epidemics of HIV, drug and alcohol abuse, and unemployment. Standard HIV prevention programs, located in healthcare settings and/or using counseling models, fail to engage men. Soccer and vocational training are examined as contexts to deliver male-specific, HIV prevention programs.

**Methods:**

Young men (*n* = 1200) are randomly assigned by neighborhood to one of three conditions: 1) soccer league (*n* = 400; eight neighborhoods); 2) soccer league plus vocational training (*n* = 400; eight neighborhoods); or 3) a control condition (*n* = 400; eight neighborhoods). Soccer practices and games occur three times per week and vocational training is delivered by Silulo Ulutho Technologies and Zenzele Training and Development. At baseline, 6 months, 12 months, and 24 months, the relative efficacy of these strategies to increase the number of significant outcomes (NSO) among 15 outcomes which occur (1) or not (0) are summed and compared using binomial logistic regressions. The summary primary outcome reflects recent HIV testing, substance abuse, employment, sexual risk, violence, arrests, and mental health status.

**Discussion:**

The failure of men to utilize HIV prevention programs highlights the need for gender-specific intervention strategies. However, men in groups can provoke and encourage greater risk-taking among themselves. The current protocol evaluates a male-specific strategy to influence men’s risk for HIV, as well as to improve their ability to contribute to family income and daily routines. Both interventions are expected to significantly benefit men compared with the control condition.

**Trial registration:**

ClinicalTrials.gov registration, NCT02358226. Registered 24 November 2014.

**Electronic supplementary material:**

The online version of this article (10.1186/s13063-018-2804-3) contains supplementary material, which is available to authorized users.

## Background

The DREAMS Initiative was launched by global donors to reduce adolescent and young women’s risks for acquiring HIV [1]. Concurrently, Option B+ is being broadly implemented to eliminate perinatal HIV transmission and increase the length and quality of the lives of women living with HIV in sub-Saharan Africa [2]. While women almost always acquire HIV from their male partners, there are few such initiatives for men nor have men been successfully engaged in HIV prevention or care [3]. This study examines a gender-specific HIV prevention strategy for South African men.

HIV incidence among young, black men in South Africa has remained stable at 3% per year [4], resulting in 2.4 million South African men infected with HIV [5, 6]. New infections occur at an older age among men compared with women [6] and are associated with a cluster of risky behaviors. For example, poly substance use is endemic in South Africa; about 30% of young men report visiting bars more than 10 times per month, 30% have symptoms of alcohol dependency, 50% have recently used marijuana, 32% use methamphetamine, and 20% use mandrax (methaqualone/quaalude, a sedative/hypnotic) [7–11]. Substance use often leads to township violence [12] and is linked to 60% of automobile accidents, 75% of homicides, 67% of domestic violence, and 30% of hospital admissions [13]. Alcohol use alone costs South Africa about 9 billion South African Rand (ZAR) annually [14]. Furthermore, about half of township men have been arrested or are in jail, 22% have been in prison, and 20% report being gang members [11]. More than a third of young men have had recent concurrent sexual partnerships, fail to use condoms [15–18], and experience repeated sexually transmitted infections (STIs) [19, 20]. Almost 30% of young men report that at some point they have forced a woman to have sex [11]. Thus, there are substantial data showing that South African men are in need of efficacious interventions to reduce their risk for HIV, and their HIV risk appears related to a cluster of risky acts.

While men experience many risks, HIV prevention resources are primarily anchored in healthcare settings—a setting underutilized by men [21, 22]. In particular, most women find out about their HIV infection in antenatal care, a setting which men are typically excluded from visiting [22]. Seropositive men seek care at much later stages of disease than women [23].

One explanation for men’s underutilization of healthcare is the incompatibility of existing intervention models with young men’s identities, roles, and preferred styles of acting [11, 24, 25]. Existing evidence-based interventions (EBI) for HIV are based on counseling models of “tend and befriend”, which are common female strategies for coping with stress [26]. In contrast, men more often utilize a “fight or flight” strategy for coping with stress [26]. Traditionally, men’s engagement in EBI for HIV has been lower than that of women [27].

Young South African men need new pathways for prosocial roles and behaviors. Concurrently, interventions need to be attractive and consistent with men’s styles. We have designed a behavioral intervention around soccer to “pull” or engage men in the intervention, rather than having to “push” public health strategies into their lives. For men, sports activities are highly attractive activities and promote a social and personal identity [28]. South Africans associate soccer with a strong sense of national identity [29] and sports elicit strong feelings of masculine identity [30]. Soccer potentially creates a replacement for the strong masculine roles in tribal society that were destroyed with rapid migration to peri-urban settlements in the Western Cape of South Africa. In the context of having fun or planning for the future, young men acquire healthy habits of daily living and reinforce positive community norms. In our pilot study, soccer was a highly desirable activity; 95% of men enrolled in the pilot and 80% were successfully engaged on a sustained basis, participating in roughly three practices and a game weekly over 6 months [11]. Even if they were not good players, the young men valued being part of the team. In qualitative interviews, families felt uniformly positive about soccer. It occupied young men, gave them respect in the community, and games were an opportunity for the community to attend and value the young men.

In addition, there is evidence that sports and physical activity are associated with reduced rates of substance abuse and HIV risk [31–33], and have been recognized as a rewarding public health strategy [34]. Similarly, sports programs promote healthy daily habits. Requiring young men in the intervention to consistently arrive on-time, attend practices, and to be drug-free mirrors the requirements for employment. The soccer intervention may establish these habits, create prosocial networks between men, and offer men opportunities to demonstrate their reliability. Their soccer networks may also provide new relationships and opportunities for linking to employment.

Linking to employment is critical. Unemployment is normative in South Africa’s townships, affecting 7 million persons annually [6, 35]. In Cape Town, 66% of young people are unemployed and many face lifetime unemployment [36]. Men have also been largely excluded from economic development programs [37, 38], even though these interventions have similar outcomes across gender [39, 40]. There are typically no community centers that engage young people, and the majority of young men stop school at 10th grade [41].

In 2000, the South African government established the Services Sector Education and Training Authority (SSETA), a job training program operated by the Department of Labor [42]. Although there is an annual budget of more than ZAR 3 billion, fewer than 0.9% of youth receiving the vocational training from SSETA are provided with any type of on-the-job training and 60% are unemployed at the completion of the program [43]. When young people are finished with job training, there is no network to find jobs and young people do not have the tools to initiate their own businesses [44]. The government offers ZAR 350 to provide apprenticeships; however, this requires about 10 h of paperwork per month by both the employer and employee to access the monthly stipend and additional tax breaks [45]. Given these challenges, apprenticeships are underutilized and SSETA funds are consistently underspent annually [45]. Interpersonal support is missing from the job training programs of the South African government; we plan to provide this through a combination of two programs—the Silulo Ulutho Technologies and Zenzele Training and Development—in the proposed intervention. Silulo Ulutho Technologies, established in 2004, is an award-winning infrastructure technology (IT) company that offers specialized training programs through their Training Academy. For the proposed project, Silulo provides accredited public computer courses. Zenzele Training and Development, based in Khayelitsha, provides training in vocational and business skills, as well as mentorship. Zenzele, which means “do it yourself” in Xhosa, was established in the late 1990s. For the proposed project, Zenzele offers accredited courses on woodwork and welding. Between the two programs, participants have opportunities to develop both the interpersonal and vocational skills required for gainful employment.

In a pilot evaluation of this intervention, we found that 30% of young men secured jobs (despite none having employment at baseline) [11]. We trained men for five different jobs: painting, electrical work, construction, mobile phone repair, and plumbing. The training was useful to some young men; however, assisting young men in job preparation or placing them in a position needs to be highly personalized. We provided clothes for job interviews, business cards, and helped young men find potential job opportunities. We are building on these interpersonal strategies in the proposed randomized controlled trial (RCT) with both the Silulo Ulutho Technologies and Zenzele Training and Development programs.

**Fig. 1:**
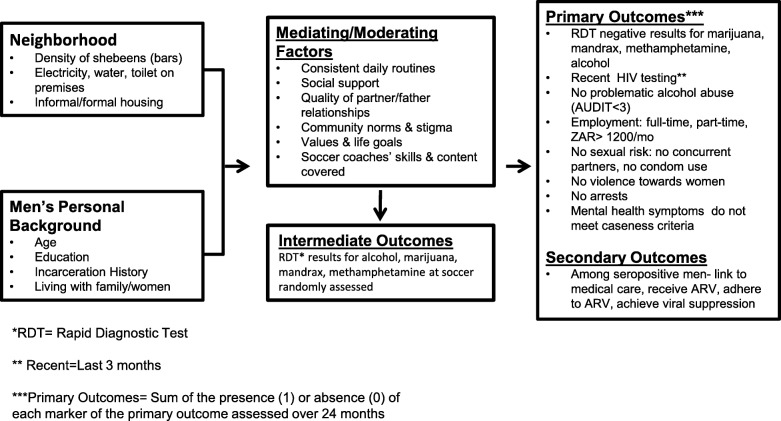
Relationships among contextual/background factors, mediating/moderating influences, intermediate outcomes, and primary and secondary outcomes. ARV antiretroviral, AUDIT Alcohol Use Disorders Identification Test, RDT rapid diagnostic tests, ZAR South African Rand

**Fig. 2:**
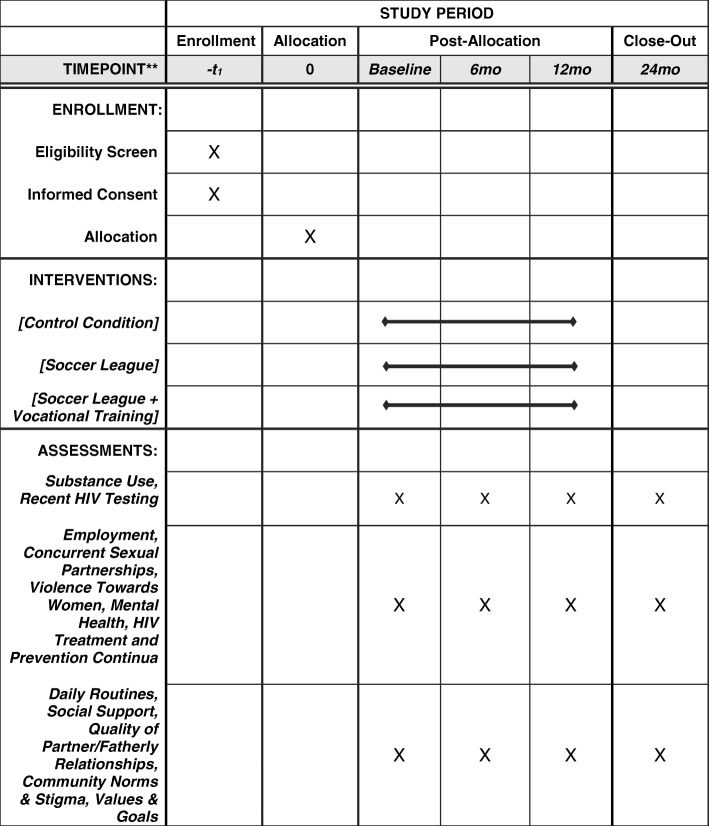
Schedule of enrolment, interventions, and assessments

This paper summarizes the protocol of the RCT being implemented in the townships of Cape Town, South Africa, to evaluate the efficacy of this strategy. Twenty-four neighborhoods are randomized to one of three conditions: 1) soccer training; 2) soccer and vocational training; or 3) a control condition. The primary hypothesis is that there will be an overall significant improvement in the number of significant outcomes (NSO) found among 15 correlated outcomes, which will be assessed over 24 months comparing the soccer, soccer plus vocational training, and the control conditions. Figure 1 outlines the underlying theoretical model and Fig. 2 shows the schedule of enrollment, interventions, and assessments (SPIRIT figure).

## Methods/design

### Ethical approvals and management structure

The Institutional Review Boards (IRB) of the University of California, Los Angeles (UCLA; IRB no.14–001587) and Stellenbosch University (N14/08/116) approved the current study protocol (Version 3.0, 1 June 2018). The IRBs approve any changes to the study protocol, and the Clinical Trials registration is updated in the event of protocol modifications. The study protocol follows the Standard Protocol Items: Recommendations for Interventional Trials (SPIRIT) and the populated checklist is provided as an Appendix (see Additional file 1). Authorship is based on standards outlined by the American Psychological Association [46].

### Data monitoring

We formed an independent Data Safety Monitoring Board, comprised of four consultants with substantial research experience in South Africa and the United States. The DSMB meets twice annually and reviews interim analyses after each round of follow-up data collection at 6, 12, and 24 months. Stopping the trial will occur only if there appears to be iatrogenic effects associated with men meeting in groups and exacerbating each other’s risk acts. In particular, we monitor criminal acts, sexual risk, and increased substance use that could be associated with the intervention at the 6- and 12-month interim analyses.

### Data management

The Data Management Team, comprised of three statisticians and a data manager based at UCLA, provide ongoing analyses to the DSMB. In addition, they are responsible for ensuring successful randomization and data analyses.

A separate Assessment Team implements all assessments, follow-up interviews, and testing, and are responsible for quality data collection. Assessments are completed at a local storefront research study space. In addition to rapid diagnostic tests (RDT) for HIV and substance use at each scheduled assessment of outcomes, men in the two active intervention conditions are randomly tested for substance use while at soccer practices and games. At the outset of the study, the Assessment Team is blinded to intervention assignment, but participants often discuss their intervention activities during assessment interviews, causing unblinding over time. An Intervention Team, with a different supervisor, implements the soccer and vocational training interventions.

### Site

**Fig. 3:**
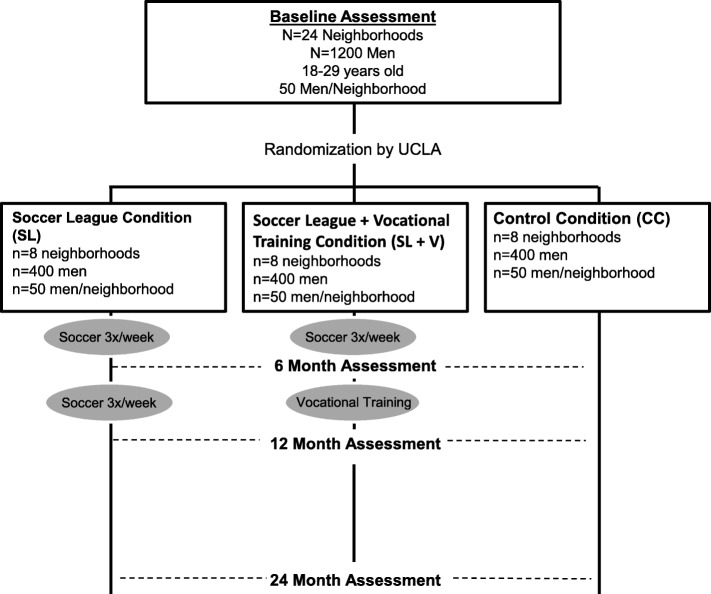
Design of the cluster randomized controlled trial

Cape Town is comprised of five major peri-urban settlements with formal and informal housing (shacks). Using charts of field workers of each community and street-intercept surveys of male residents, UCLA matched 24 neighborhoods based on the recency of migration, the density of bars (shebeens), the percentage of informal housing, and water on the premises. Buffer areas of at least 1 km or natural barriers, such as highways, railways, and rivers, separate each neighborhood. Contamination is further prevented by young men’s lifestyles, which lack readily available transport and are concentrated at kiosks, shebeens, and churches in their local areas. In each area, there is formal housing and vast areas of informal shacks. Each neighborhood contains approximately 450–600 households, including roughly 50 young men aged 18–29 years old. We have identified additional neighborhoods as back-up recruitment sites in case a natural disaster or political problem forces exclusion of any triad from our study. Following recruitment, the Data Management Team randomizes triads using R [47], and is not blinded to intervention assignment. Figure 3 summarizes the design of the cluster randomized controlled superiority trial and participant flow.

### Recruitment

Recruiters underwent 1 month of training. Recruiters go dwelling-to-dwelling, randomly selecting the first household (by flipping a coin on a hardcopy neighborhood map, with a supervisor) to enter and then systematically approach houses in concentric circles until 50 young men aged 18–29 are identified per neighborhood and agree to participate, documenting all refusals. To be considered eligible, young men must be aged 18–29, have slept at least four nights per week in the household for the 2 months prior to recruitment, speak Xhosa or English, do not appear to be under the influence of any substances at the time of recruitment, and are able to understand the recruiter. Any potential participant who does not meet the five inclusion criteria defined above is considered ineligible and is not invited to participate in the study. We anticipate that it may take several household visits to contact and recruit the young men identified by their family members on recruitment visits. Since housing units are typically too small to provide confidential interviewing, we transport young men to a local storefront research study space to complete assessments. Recruiters remain in constant contact with drivers, so that when a potential participant expresses interest, the driver can both confirm that they live within the catchment area and transport them to the local storefront assessment center.

### Assessments

At the local storefront assessment center, trained interviewers obtain written voluntary informed consent and then administer a 1-h assessment, recording responses on mobile phones using a program designed by Mobenzi Technologies. Participants receive about ZAR 150 for their time to complete the interview (incentives are adjusted over time for inflation). Assessment data are coded with personal identification numbers (PIDs) and do not include any personally identifiable information. De-identified assessment data are available through a password-protected, cloud-based database and accessed by UCLA and Stellenbosch University for quality control and later analyses. Assessments are conducted at the baseline recruitment and at 6, 12, and 24 months later. Interviewers report all adverse events to their supervisors in real time and identify these as study-related (or not). Serious adverse events (death, criminal acts) are reported to all Investigators, Institutional Review Boards, and funding agencies within 48 h.

At the time of enrollment, interviewers collect detailed locator information from participants, including the names and addresses of friends and family members and favorite hangout spots, locally and in the Eastern Cape, where the ancestral homes of many Cape Town residents are located. The Assessment Team, led by Stellenbosch University, is experienced in using this information to retain participants, consistently maintaining participant retention rates at or above 85% [48].

### Outcome measures

The primary outcome is the NSO out of 15 outcomes, as explained by Harwood et al. [49]. The 15 specific outcomes are explained below, along with how we intend to analyze each outcome. We analyze each outcome separately. We count the number of outcomes out of 15 where the treatment effect is significant at *p* = 0.05 (two-sided) and that count is the NSO. The single NSO outcome allows us to identify the outcomes affected by the intervention while adjusting for multiple comparisons and the correlations among the outcomes. For fifteen outcomes, the NSO needed for a significant result is either 2 or 3 outcomes, depending on the correlation among the outcomes. As a practical matter, the cutoff is 2 only if the correlation among outcomes is near 0 or near 1. For correlations among outcomes between 0.1 and 0.9 (see Table 2 of Harwood et al. [49]), three significant outcomes give significance at the 0.05 level and, based on similar work with similar variables in these same neighborhoods of Cape Town, we plan to require a minimum of three significant outcomes to declare that the treatment is effective.

#### Fifteen outcomes

We have 15 primary outcomes: 1) Alcohol Use Disorders Identification Test (AUDIT-C) score < 3 (i.e., problematic alcohol use); 2) no alcohol usage in last 24 h; 3) PEth Alcohol Test (excessive alcohol use in prior 3 weeks, at 24 months only); 4) no marijuana (dagga) usage in the last 10 days assessed via RDT; 5) no quaalude (mandrax) usage in the last 2–3 days assessed via RDT; 6) no methamphetamine (tik) usage in the last 1–2 days assessed via RDT; 7) HIV testing in the last 3 months; 8) employment (part/full-time); 9) income above 1200 ZAR/month; 10) no concurrent partnerships; 11) no sex without condoms; 12) no violent acts toward women; 13) no arrests by police; 14) engaged in a community activity; and 15) Center for Epidemiologic Studies of Depression (CES-D) score < 16 (i.e., caseness). Each primary outcome measure is a binary variable (not present or present).

#### Substance abuse

A combination of rapid diagnostic tests (RDT) and the AUDIT-C [9, 50, 51] assess substance abuse. The RDT includes the REDLINE Breathalyzer (determines alcohol use in the last 24 h), the PEth Alcohol Test (assesses excessive alcohol use in the last 3 weeks, at 24-month assessment only), and urine tests for marijuana, methamphetamine, and mandrax which document the alcohol or drugs as present (0) or not (1). The AUDIT-C is a brief, reliable and valid, three-item questionnaire of problematic alcohol use for which the time frame was adapted to shift from 12 months to 6 months. The three questions, each with a range of 1 to 4, cover the frequency of: 1) days of any alcohol consumption; 2) usual number of drinks daily; and 3) days on which > 6 drinks are consumed. The AUDIT-C demonstrates good sensitivity and specificity at a cut point of 3 or greater for identifying risky drinking (0) or not (1) and performs well in South Africa [9].

#### Recent HIV testing

Recent HIV testing is self-reported as occurring (1) in the last 3 months or not (0).

#### Employment and income

Employment is measured using self-reports of the number of jobs held in the last year. Employment occurs (1) or not (0). Income is self-reported income above 1200 ZAR/month (1) or not (0).

#### Concurrent sexual partnerships and condom usage

Concurrent sexual partnerships are self-reported as having sexual partnerships overlapping in occurrence in the last 3 months (0) or not (1). Condom usage is always using condoms in the past 3 months (1) or not (0). No sex counts as (1).

#### Violence toward women

Violence against women is self-reported as threatening or perpetrating abusive behavior (physical and sexual; 0) or not (1).

#### Arrested

Self-reported arrest in the past 3 months as yes (0) or no (1).

#### Community activity

Self-reported as engaged in a community activity (1) or not (0).

#### Mental health

Mental health is evaluated by 20 items, each with a response range from 0 to 3, reflecting endorsement of symptoms of depression on the CES-D scale [47]. The scale has been found reliable (alpha > 0.85) in previous research [48]. Participants with a CES-D score greater than 16 are considered to have a depressed mood (0) and those < 16 are not (1).

### Secondary outcomes

#### HIV prevention and treatment continua

Among seronegative participants, self-reports document the utilization of repeat HIV testing, access of medical care, discussions of HIV prevention with sexual partners, and disclosure of serostatus. Among participants living with HIV, participants self-report engagement in medical care, uptake and adherence to antiretroviral (ARV) medications, and medical regimens over time. HIV testing is offered at the soccer fields by a mobile testing van and uptake is documented. Given the low self-reported rate of HIV positivity, we ask if participants want to share results as they test in real time at the 6-, 12-, and 24-month assessment. The key comorbidities affecting the HIV continua are included in the fifteen study outcomes (e.g., mental health symptoms, substance abuse, violence toward women).

#### Intermediate, mediating, and moderating factors

Consistency of daily routines: Sustaining a job requires consistent daily routines of waking up, going to work at the same time, being ready for work, preparing food, sleeping, etc. Interviewers ask young men to report their daily routines in the last 3 days: the number of meals eaten with others, time of going to bed and waking up, and whether household chores are done.

Density and centrality of social support networks: Similarly, interviewers ask the young men to list their friends and identify how often support (instrumental, emotional) is given or received, and the consequences of support received [52].

Quality of relationships with partner and father: These relationships are monitored from young men’s reports and include questions on their household members, allocation of household tasks, income contributions, discussion of health status, and mealtime involvement.

Community norms around HIV stigma, alcohol use, healthy nutrition, and hopefulness: Given HIV stigma is a major barrier to timely HIV testing and linkage to care, interviewers ask whether HIV is considered a punishment and whether it should or should not affect their treatment in society.

Values and goals: Participants’ values, life goals, and goals for the next month are assessed during assessments at 12 and 24 months.

### Intervention

All young men in a neighborhood are assigned to the same condition. Participants will be removed from the study if they directly threaten or are physically violent toward any study staff or other participants, or if they are seriously disruptive or verbally abusive to peers or staff during soccer training, a soccer game, or assessment at least three times in a 2-week period. On a weekly basis, participants in the intervention conditions are tested for substance use. Any participant with a positive drug or alcohol screen is barred from attending the intervention that day.

#### Condition 1: control condition (CC)

Following the baseline interviews, young men in the eight neighborhoods randomized to the control condition (*n* = 400 men) routinely receive flyers with picture stories regarding HIV prevention strategies and how to access these strategies locally: HIV testing, circumcision, HIV treatment including ARV, condoms, and STI testing and treatment. These young men are only contacted for assessments 6, 12, and 24 months later following the baseline interview.

#### Condition 2: soccer league (SL)

All young men in eight neighborhoods (*n* = 400) receive soccer training for 1 year. After all of the baseline assessments are completed in a specific neighborhood, soccer coaches visit each participant individually to describe the intervention in detail, invite them to the soccer league, and answer any questions. The league includes at least two and usually three teams per neighborhood (14 men minimum per team needed, anticipating some absenteeism), resulting in approximately up to 24 teams in this condition. Each week, there are 2 days of practice and 1 day for a game with another team. Teams are led by coaches who are recruited from the broader township (versus target neighborhoods) to safeguard players’ confidentiality (coming from the same neighborhood is likely to inhibit men’s disclosures about problem issues, fearing the coach may tell others). Coaches cannot be regulars at the local shebeens or known drug addicts, and must have held a job at some time previously, have experience playing soccer, and demonstrate good social skills. In this way, coaches are positive peer deviants (i.e., role models) for participants [53, 54]. About 30% of persons applying to be coaches are hired, based on initial interviews and observations completed during the coach training.

**Fig. 4:**
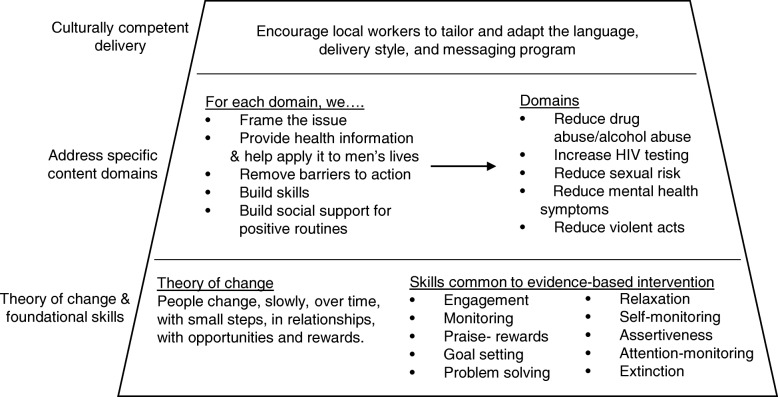
Model reflecting the training program implemented with coaches to deliver the HIV preventive intervention

Figure 4 summarizes the training model for intervention delivery. We do not ask coaches to replicate a manual with fidelity. We train all coaches in the foundational skills and theory common across evidence-based psychotherapeutic interventions [55, 56] and adolescent HIV prevention programs [57], the bottom tier on Fig. 4. Coaches are then trained in specific content areas (i.e., the key messages to be given in the intervention), the middle tier in Fig. 4. This includes training in life skills designed around six topics (reducing alcohol/drug use, increasing HIV testing, routinely getting health check-ups and optimizing interactions with healthcare providers, creating enjoyable and healthy daily routines, building friendship networks that are not based on shared risk behaviors, and managing money (real cost of alcohol/drugs)). Each of these topics is repeatedly rehearsed with role-play during training so that coaches practice delivering health messages. Coaches also practice brief alcohol interventions, and how to acquire information about HIV, tuberculosis (TB), alcohol use, and employment. To better facilitate referrals, coaches visit local clinics to meet their staff and learn about the services provided. Each coach also receives training in an EBI, the Street Smart program, which is a 10-session, small-group intervention for high-risk youth [58–60]. This EBI has been adapted for Uganda and South Africa [61]. Coaches do not replicate the delivery of this intervention with young men, but the training provides a demonstration model for an EBI. HIV EBI share common processes, components, and principles [57, 62]. These procedures are repeatedly reviewed over the entire training program, so that the coaches are very familiar with HIV and substance use prevention and cognitive-behavioral shaping. However, coaches are allowed flexibility in implementing specific activities. Coaches report when, which, and how the practices are used to initiate topics regarding HIV and substance use via mobile phones daily.

Coaches also receive 1 month of training that covers IRB certification and human subject training, as well as how to report all conversations, contacts, and their content daily on the mobile phone system. Coaches attend a weekly supervision meeting to review progress, and reinforce key skills and content. In-service training occurs routinely on a monthly basis.

#### Process measures for soccer

Using a mobile phone survey programmed by Mobenzi at each practice or game, coaches report on: attendance, whether HIV, alcohol and drug RDT are administered, the content area discussed during the coaching meeting (e.g., alcohol use, sexual relationships), and the skills used by the coach. Stellenbosch supervisors randomly visit either practices or games once every 2 weeks to provide on-site supervision and monitoring. HIV testing is offered at the soccer field by a mobile testing van and uptake is documented (but not the test results). RDT for alcohol, marijuana, methamphetamine, and mandrax are administered randomly once per week to all participants.

#### Condition 3: soccer league and vocational training (SL-V)

For the first 6 months of intervention delivery, all procedures described above for the SL condition are replicated for all young men in eight neighborhoods (*n* = 400 men). The selection, training, and monitoring system for coaches and participants is the same in both conditions for the first 6 months, with soccer offered 2 days per week and a game on Saturdays.

After 6 months, all young men are offered vocational training through either Silulo Ulutho Technologies or Zenzele Training and Development programs. In these programs, participants have access to accredited training programs in public computer courses, woodwork, and welding. Both programs are located in Khayelitsha, which is close to participants’ homes, thus avoiding transport-related barriers. Additionally, the training programs occur in a mentor-mentee context so that participants can develop the interpersonal skills required for employment.

### Data analysis

Histograms for continuous variables (CES-D, AUDIT-C) will be plotted and screened for outliers and incorrect data and we will build tables for categorical variables, marginally and by treatment condition. Longitudinal data analyses follow the general analysis plan described in detail in Weiss [63]. Longitudinal data will be plotted for continuous variables and empirical summary plots (means ± 2 SE over time, by treatment condition with means and SEs calculated simply at each time point within condition) for all variables.

Neighborhoods and soccer teams are nested within condition; we will use hierarchical regression models with random effects for neighborhood, team within neighborhood, and young men. However, for many outcomes, one or other of the random effects variances will likely be small. Traditional maximum likelihood (ML) software has trouble fitting random effects models when random effect variances are modest or small. However, it is important to include these random effects in the model and to have non-zero variance estimates to properly assess the evidence in the data about the conditions, no matter the size of the variances. Working with SAS or Stata and having to specially adapt models to every outcome due to ML neighborhood and team variance estimated as zero is expensive. The simple but flawed solution of setting the variances to zero causes *p* values comparing conditions to be inappropriately smaller than they merit. In contrast to ML methods, Bayesian methods will not set variance estimates equal to zero, and will properly fit the models we need to our data. We will use the software MCMCglmm [64] or JAGS in R [65], as they both can fit the needed generalized linear mixed models (GLMMs) for discrete and continuous outcomes, estimate all variances appropriately, and run without producing errors, thus saving substantial time and improving the accuracy of the resulting conclusions. To calculate the two degrees of freedom chi-square statistic, we will calculate the posterior mean and covariance matrix of the difference of differences SL-V minus CC, and SL minus CC at 24 months minus baseline and calculate a two degrees of freedom chi square statistic for which we calculate a tail area. The 24-month outcome will be the main time point, with secondary calculations for 6 months minus baseline and 12 months minus baseline.

### Missing data

We will analyze: 1) missing data longitudinally as a binary outcome at 6, 12, and 24 months, omitting the first time point; 2) separately, we will analyze which men drop out as a single binary outcome. Baseline sociodemographic variables (e.g., age, education) will be examined as predictors in each analysis; and 3) we will use intervention conditions as predictors of baseline sociodemographics. Any significant variables (analyses 1 and 2) and any sociodemographics that are predicted by condition (analysis 3) will be collected together and used as additional predictors in sensitivity analyses when assessing significance of the conditions.

Additional information is available as counts for several outcomes (e.g., number of days of drug use, number of partners) and continuous variables (CES-D, AUDIT) which we will analyze in secondary analyses. Among the subset of HIV-positive participants, we will analyze the HIV treatment continuum variables (i.e., linkage and retention in care, uptake and adherence to ARV). For binary outcomes measured at baseline and 6, 12, and 24 months, we expect to treat time categorically, and model main effects for condition, time, and time by condition interaction. Primary interest is the differences among conditions in the change from baseline to 12 months and to 24 months, and secondarily the change to 6 months. Assuming significant differences between conditions in changes from baseline, we will estimate pairwise differences (SL-V minus SL; SL minus CC; SL-V minus CC) in changes from baseline to months 6, 12, and 24.

Finally, we will summarize the frequency of practice elements delivered by coaches from process monitoring data in tables overall and by coach. We wish to see if supervisor ratings are predictive of practice element data that we collect; the model is longitudinal repeated measures but with coaches as the subject. Also, using GLMMs, we will analyze the process measures that are measured regularly (daily/weekly/monthly) during the soccer intervention to compare SL and SL-V conditions and to see how they relate to future employment. We expect that coaches who most frequently use goal setting and problem solving will have the young men with the greatest improvements. One year after the completion of data collection, a data set stripped of potential identifiers will be provided to the National Institutes of Health and available at UCLA.

Sample size calculations were carried out in two steps, similar to our previous studies in this area [66], to achieve 80% power with a 0.05 alpha level. First, calculations were carried out to compare two conditions (e.g., SL-V versus SL) on a dichotomous outcome, since dichotomous outcomes generally require larger sample sizes than continuous outcomes. Using employment as an example, we assume an employment rate of 30% at 12 or 24 months in one of the conditions. The sample size will achieve sufficient power if the other condition has 17% (or less) or 41% (or more) employment. We anticipate actual employment differences between groups bigger than 13% (= 30% – 17%) or 11% (= 41% – 30%). We assume 20% loss to follow-up (the pilot had 5% loss at 6 months). This sample size calculation estimates the total sample size for two groups. The additional sample needed for the third group is simply the total sample size divided by two. As a second step, the total sample size is inflated to account for the double nesting of men within teams and teams within neighborhoods. We apply the design effect adjustment (1 + (*n* – 1) × rho) twice, where *n* is the cluster sample size (17 participants per team at the most, and 50 participants per neighborhood) and we take the intraclass correlation (rho) to be 0.01, consistent with previous studies in these neighborhoods [66]. The design effect for our study is the product of these two effects, 1.49 × 1.16 = 1.7284. The CC condition has less design effect than the SL and SL-V conditions, but we have conservatively adjusted the calculations for both design effects across all three conditions; alpha = 0.05, power = 0.8. After carrying out the second step, we arrive at our target sample size of 1200 participants, with 400 per condition. As we will have longitudinal data on employment, our analysis will actually offer smaller standard errors of estimation, and thus we expect to have even greater power than this. In our pilot, 40% of young men had used hard drugs [11]. Comparing two conditions, we have power to detect a decrease to 26% in SL or SL-V from CC. Since for drug use and other analyses we get to adjust for baseline hard drug usage, the power improves substantially.

### Publications and community involvement

Dissemination of findings will be provided to the community through meetings, which will serve as an opportunity for researchers to discuss results and receive feedback from community members. Publications will also be produced regularly and made publicly available in accordance with the National Institutes of Health’s Open Access Policy.

## Discussion

This intervention has several important innovations. With soccer as a medium, coaches are initiating discussions to influence community norms about HIV, alcohol and drug use, and employment. By delivering this health messaging, coaches serve a similar role as paraprofessional community health workers (CHWs), which are considered to be key resources for broadly diffusing EBI. However, health-based CHWs are almost always women, typically delivering EBI to other women [67–69]. Not only does this study represent one of the few HIV interventions in South Africa that targets men, but it is also novel in that it concurrently addresses several of the main structural challenges faced by township men, with the goal of improving their health outcomes as well as their social relationships and employability. Furthermore, the intervention is delivered in a highly attractive setting for men—playing soccer. In the past, shebeen-based interventions have been successful in reducing risk for HIV over 6 months [9]. This soccer-based intervention provides the same opportunities, and could also potentially replace the pattern of having shebeens as the center of men’s social relationships. The second component of the intervention, vocational training, facilitates an important pathway to finding a job, a top priority in these areas facing high unemployment [36].

While working in townships and outside of clinical settings may help engage high-risk men in the intervention, it also creates challenges and limitations. Because the intervention is largely carried out on the soccer field rather than in a clinic or university, many resources (i.e., nurses and physicians, treatment, counseling, and laboratory testing facilities) are not available onsite. Furthermore, as Dishion et al. and Kaminer have demonstrated, risky young men can serve to disinhibit the harmful and delinquent behaviors of peers in group interventions [70, 71], particularly if most of the young men already engage in high-risk behaviors such as substance use. These challenges are addressed in multiple ways. Testing for HIV and substance use is easily conducted outside the laboratory by coaches and assessors using RDT with mobile integration. To combat risky group behavior, coaches serve as role models who can influence young men by example and share their normative beliefs around HIV, alcohol and drug use, and the daily routines that protect against risky behaviors. Diffusion of innovation theory and our experience with popular opinion leader interventions both posit that 15% of the group behaving as positive peer leaders is enough to diffuse positive norms and build toward a tipping point [72, 73]. Coach training includes specific strategies to combat the development of norms increasing risky behaviors among the men in addition to behavioral management strategies, and familiarity with important resources for referral as needed. A month of full-time training for coaches is considerable and, some argue, non-replicable. Our proposed coach training is a similar length to that of the broadly-scaled CHW training through the Philani Maternal, Child Health and Nutrition Project [66]. However, the coaches are able to contact 28 young men daily (with two teams of at least 14 men each), not the typical four home visits per day by CHWs.

Reaching young men in South African townships, this study contributes to the existing literature on HIV intervention strategies in multiple ways. Many donor agencies have mobilized to reduce HIV incidence among young women in South Africa. However, young men are the primary source of the infection of young women and the rate of HIV infections of men increases over time [74]. A great deal of energy has been expended to create gender-tailored strategies to reduce HIV risk among young women. This protocol focuses on a parallel gender-tailored strategy to reduce HIV among young men by addressing the structural conditions that increase HIV risk (e.g., unemployment and high rates of substance use). If successful, this intervention offers a cost-effective model for engaging young men in healthcare and improving their health and economic outcomes in South Africa and other low-resource settings.

### Trial status

At the time of manuscript submission, recruitment for the study is underway but has not been completed.

## Additional file

Additional file 1: Soccer for health SPIRIT checklist. (DOC 125 kb)

## Abbreviations

ARV: Antiretroviral
AUDIT-C: Alcohol Use Disorders Identification Test
CC: Control condition
CES-D: Center for Epidemiologic Studies of Depression
CHW: Community health worker
DSMB: Data Safety Monitoring Board
EBI: Evidence-based interventions
GLMM: Generalized linear mixed model
ML: Maximum likelihood
NSO: Number of significant outcomes
RCT: Randomized controlled trial
RDT: Rapid diagnostic tests
SL: Soccer league
SL-V: Soccer league and vocation training
SSETA: Services Sector Education and Training Authority
STI: Sexually transmitted infection
